# Characterization of Tissue-Engineered Posterior Corneas Using Second- and Third-Harmonic Generation Microscopy

**DOI:** 10.1371/journal.pone.0125564

**Published:** 2015-04-28

**Authors:** Louis Jay, Jean-Michel Bourget, Benjamin Goyer, Kanwarpal Singh, Isabelle Brunette, Tsuneyuki Ozaki, Stéphanie Proulx

**Affiliations:** 1 Centre Énergie Matériaux Télécommunications, Institut National de la Recherche Scientifique, Varennes, Quebec, Canada; 2 Centre de recherche de l’Hôpital Maisonneuve-Rosemont, Montréal, Quebec, Canada and Département d’ophtalmologie, Université de Montréal, Montréal, Quebec, Canada; 3 Axe médecine régénératrice, Hôpital du Saint-Sacrement, Centre de recherche du CHU de Québec, Québec, Quebec, Canada and Centre de recherche en organogénèse expérimentale de l’Université Laval / LOEX, Québec, Quebec, Canada; 4 Département d’ophtalmologie et d’oto-rhino-laryngologie, Faculté de médecine, Université Laval, Québec, Quebec, Canada; Tufts University, UNITED STATES

## Abstract

Three-dimensional tissues, such as the cornea, are now being engineered as substitutes for the rehabilitation of vision in patients with blinding corneal diseases. Engineering of tissues for translational purposes requires a non-invasive monitoring to control the quality of the resulting biomaterial. Unfortunately, most current methods still imply invasive steps, such as fixation and staining, to clearly observe the tissue-engineered cornea, a transparent tissue with weak natural contrast. Second- and third-harmonic generation imaging are well known to provide high-contrast, high spatial resolution images of such tissues, by taking advantage of the endogenous contrast agents of the tissue itself. In this article, we imaged tissue-engineered corneal substitutes using both harmonic microscopy and classic histopathology techniques. We demonstrate that second- and third-harmonic imaging can non-invasively provide important information regarding the quality and the integrity of these partial-thickness posterior corneal substitutes (observation of collagen network, fibroblasts and endothelial cells). These two nonlinear imaging modalities offer the new opportunity of monitoring the engineered corneas during the entire process of production.

## Introduction

The cornea comprises three main layers: the epithelium, the stroma and the endothelium. The corneal endothelium is the inner most layer of the cornea, facing the anterior chamber of the eye. Its main role consists in maintaining corneal deturgescence in order to keep the corneal stroma thin and transparent. Any loss of functionality of the endothelial layer results in stromal edema and vision loss. Non-reversible corneal edema due to corneal endothelial failure accounted for 40% of the 66,305 corneal transplantations performed in 2013 in the United States [1]. Descemet stripping automated endothelial keratoplasty (DSAEK) has become the preferred technique for the surgical replacement of a diseased corneal endothelium. It implies removing the endothelium and its underlying basement membrane (Descemet membrane) and replacing it with a thin layer of posterior cornea cut from an eye bank donor eye [2]. With the increasing demand for corneal tissue and aging of the population, a shortage of donor corneas suitable for transplantation is anticipated [3, 4]. Recent progress in tissue engineering may offer new therapeutic solutions for the replacement of a diseased corneal endothelium [5, 6].

Tissue engineering now allows reconstruction of the posterior cornea. The approach used in this study takes advantage of the ability of the corneal stromal fibroblasts to secrete and assemble their own extracellular matrix to generate a corneal stroma made of fibrillar collagen, such as type I collagen. Corneal endothelial cells were seeded on top of these thick sheets of stromal substitutes, thus producing a tissue-engineered posterior cornea. This method, called the self-assembly approach, has been used for the tissue engineering of skin [7, 8], blood vessels [9–11], anterior corneas [12, 13] and three-layer corneas [14]. It offers the advantage of producing a biocompatible and potentially completely autologous tissue, without the addition of exogenous materials.

In the field of tissue engineering, there is a strong need for an imaging modality that would allow observation of the tissue at the macroscopic and microscopic scales, while preserving its structural and functional integrity [15]. Histology, immunostaining and electron microscopy are limited by fixation, dehydration and staining, which irremediably affect tissue viability, consequently resulting in the interruption of the tissue engineering process. Furthermore, three-dimensional in depth visualization of the tissue is not possible with these modalities.

Second-harmonic generation (SHG) [16] and third-harmonic generation (THG) [17] microscopy are two techniques of nonlinear optical microscopy that allow imaging of thick, unfixed, and unstained living tissues. SHG microscopy allows visualization of structures with a non-centrosymmetric molecular arrangement. The method has been well characterized [18] and is suitable for the imaging of ordered structural proteins, such as fibrillar collagens. Since the corneal stroma is primarily composed of type I collagen, researchers have used SHG microscopy to study the cornea [19–23]. THG are emitted by inhomogeneities of the refractive index or of the third-order nonlinear susceptibility [24], and are interesting tools for cell imaging [25, 26]. Simultaneous capture of both SHG and THG signals provides high spatial resolution image (because of its nonlinear optical nature) of the extracellular matrix structural organization and cellular morphologies in their native states. The benefit of these two complementary nonlinear methods was illustrated by our study on native porcine cornea, showing clear identification of the different corneal layers, including the posterior layers [27]. As in the present study, we used an Yb:KGW oscillator (1030 nm wavelength), which is particularly interesting since both harmonics fall in the visible or near ultraviolet spectra and are easily collected by standard optics. This laser also reduces the risks of photo-toxicity, since there is no autofluorescence in this wavelength range, and it generates good harmonic signals, since harmonics signals are weakly dependent on the excitation wavelength.

In this study, we characterized tissue-engineered posterior corneas using SHG/THG microscopy and compared the results with conventional histopathology to assess the usefulness of harmonic imaging in corneal tissue engineering. We demonstrated that SHG/THG imaging allows the observation of engineered cornea components with similar or better quality than traditional invasive methods.

## Materials and Methods

### Ethics statement

This study was conducted according to the guidelines of the CHU de Québec Research Center (Québec, QC, Canada) and the Declaration of Helsinki. The project was approved by the ethics committee of the CHU de Québec Research Center (#DR-002-1383). Normal human corneas unsuitable for transplantation were obtained from our local eye bank (Banque d’yeux du Centre Universitaire d’Ophtalmologie (CUO), Québec, QC, Canada). Next of kin consent was obtained from the eye bank for all the tissues provided for research.

### Tissue engineering of a posterior cornea using the self-assembly approach

Normal human corneas unsuitable for transplantation were obtained from our local eye bank (Banque d’yeux du CUO). Human corneal fibroblasts were isolated from a donor corneal stroma using an explant method and cultured as previously described [28, 29]. Ninth passaged cells were used in this study. For the engineering of the stromal sheets, cells were seeded in 25 cm^2^ flasks (BD Biosciences, Mississauga, ON, Canada) and cultured in Dulbecco’s Modified Eagle’s Medium (Invitrogen, Burlington, ON, Canada) supplemented with 10% fetal bovine serum (Hyclone, Logan, UT) and 50 μg/ml ascorbic acid (Sigma-Aldrich, Oakville, ON, Canada). Ascorbic acid allows fibroblasts to secrete and assemble their own extracellular matrix, resulting in the formation of thick sheets of collagen fibrils [30]. After 35 days of culture, two sheets were superimposed in order to produce a corneal stroma [31].

**Fig 1:**
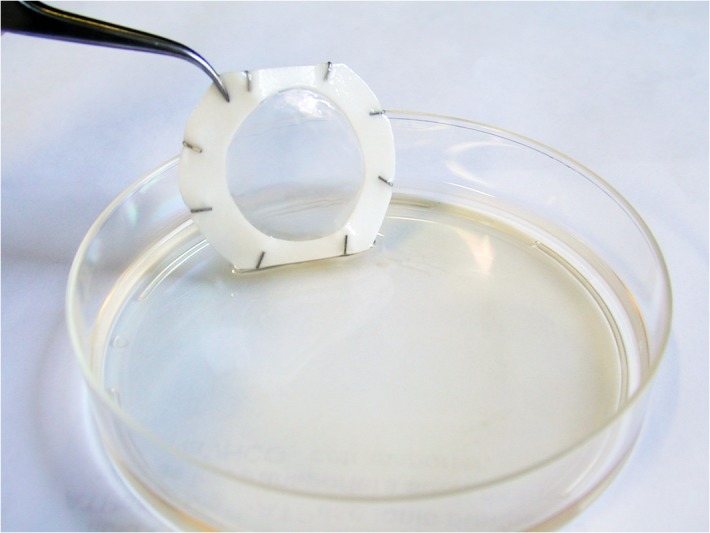
Macroscopic photograph of a tissue-engineered posterior cornea (Color version available online).

Isolation and culture of the corneal endothelial cells were performed as previously described in Zhu and Joyce [32]. The endothelium of three donor eyes (aged 11 days, 11 months and 26 years-old) was harvested. Fourth passaged cells were used in this study. Corneal endothelial cells were seeded on top of the self-assembled stromal substitute and cultured for 14 days. Three posterior corneas (example shown in Fig 1) were produced in duplicate (total n = 6). Control tissue-engineered posterior corneas were also produced without endothelial cells (n = 2). For SHG/THG imaging, three posterior corneas and one control cornea without endothelial cells were placed in a balanced salt solution (Alcon Canada, Mississauga, ON, Canada) at room temperature.

### SHG/THG imaging set-up

The experimental setup used in this work was similar to that described in Jay *et al*. [27], with minor modifications. Briefly, the laser source was a femtosecond Yb:KGW oscillator (t-Pulse, Amplitude Systèmes, Pessac, France) that delivered 200 fs pulses at 1030 nm wavelength, with a repetition rate of 50 MHz and an average power of 2 W. The average power at the sample position was about 50 mW. For both SHG and THG signals, cross-sectional observations were made by epidetection and *en-face* observations were made in transmission. The focusing objective was a water immersion objective (LUMplanFL 60×/numerical aperture 0.9/Working distance 2 mm, Olympus, Tokyo, Japan) and the collecting objective for transmission was a dry objective (Zeiss Plan NeoFluar 63×/numerical aperture 0.75/Working distance 2 mm, Carl Zeiss Microscopy GmbH, Jena, Germany) with coverslip correction. Both signals were split by a dichroic mirror, filtered and detected using photomultiplier tubes (PMT H9305-04, Hamamatsu, Hamamatsu City, Japan). Two different scanning systems were used for image acquisition. For cross-sectional imaging, motorized XY stages ensured transversal motion (normal to the optical axis Z) of the specimen and a micrometer was used for motion along the Z-axis (Micos GmbH, Eschbach, Germany). For *en-face* observations, optical scanning was achieved using galvanometer mirrors (Nutfield Technology, Hudson, NH). Motion and signal acquisition were managed using a homemade program developed with LabVIEW (National Instruments, Austin, TX).

### Histopathology

SHG and THG microscopy was performed on one of the three pairs of tissue-engineered posterior corneas and one of the control cornea without endothelial cells. The other corneas were either used fresh for alizarin red staining of the endothelium, or fixed in 3.7% formaldehyde (ACP Chemicals, Montréal, QC, Canada) for *en-face* observation with immunofluorescent staining or for histology.

For the detection of type I collagen, the main type of fibrillar collagen represented in the engineered posterior cornea, an indirect immunofluorescence assay was performed on the formaldehyde-fixed tissue. The primary antibody was an anti-human collagen type I (Calbiochem, Montréal, QC, Canada) and the secondary antibody was a goat anti-mouse IgG H+L conjugated with Alexa 594 (Invitrogen). Negligible background was observed for controls (primary antibodies omitted). Fluorescence was observed by epifluorescence microscopy (Eclipse E6000; Nikon, Mississauga, ON, Canada) and slides photographed with a numeric charge-coupled device camera (Sensys; Roper Scientific, Trenton, NJ). Alizarin red staining was performed as described in Taylor and Hunt [33], except that trypan blue was omitted. For histology, Masson’s trichrome staining was performed on 5 μm sections. Slides were observed by light microscopy (Eclipse E600; Nikon) and photographed (Coolsnap, Roper Scientific Photometrics, Tucson, AZ).

### Thickness measurement

Thickness of the tissue-engineered posterior corneas and of control cornea without endothelium was measured on histology cross-sections (1 count/picture, 3 to 7 pictures/engineered cornea) using the Axiovision Software (Carl Zeiss Microscopy GmbH). Thickness measurements were also obtained using SHG/THG 1D scan (S1 Fig) perpendicular to the tissue surface (4 to 10 measurements/engineered cornea) using IGOR Pro (WaveMetrics Inc., Lake Oswego, OR). Mean thickness values and standard deviations are reported.

## Results

### Macroscopic aspect and cross-sectional observations of the tissue-engineered posterior cornea

**Fig 2:**
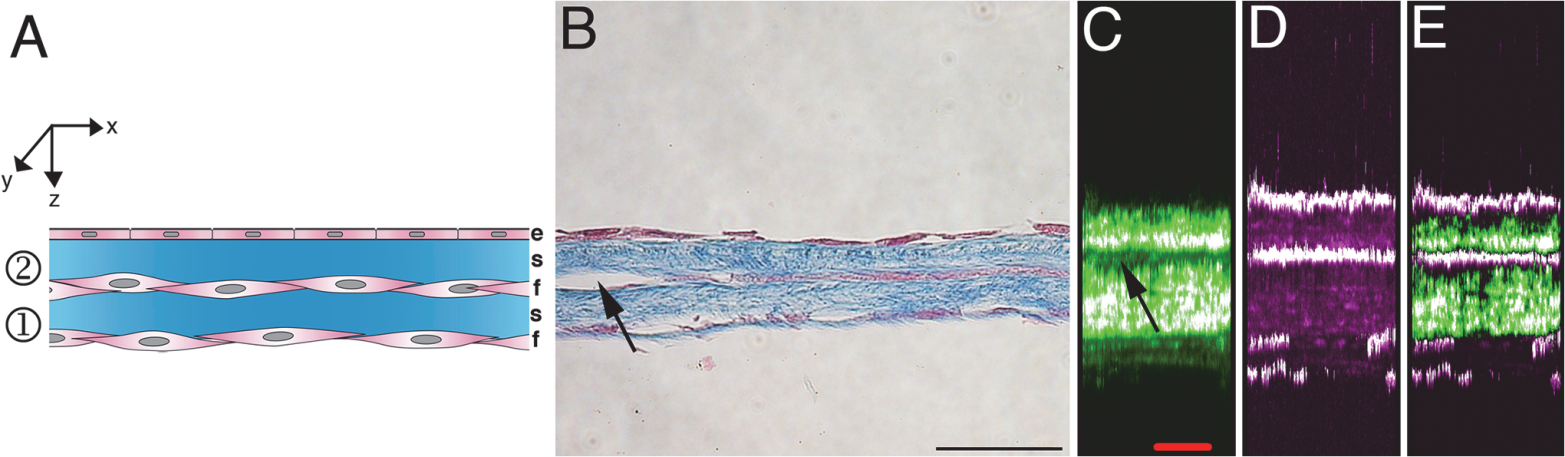
Cross-sectional observations. A) Schematic representation of the tissue-engineered posterior cornea. The tissue-engineered posterior cornea comprises a monolayer of endothelial cells adherent to the first of the two sheets of self-assembled corneal stromal matrix. B) Histology cross-section (Masson’s trichrome staining). This staining gives a purple coloration to cells and a blue coloration to collagen. Note that the endothelium forms a monolayer on top of two sheets of the tissue-engineered corneal stromal matrix. C) SHG imaging. D) THG imaging. E) Merge of SHG (green) and THG (purple). SHG/THG imaging highlights the same structures as histology, without fixing the tissue. Note that histology (B) and SHG/THG images (C-E) are two representative images (not the same region). Arrows in B) and C) show a small region where the two sheets are detached. ① = first sheet, ② = second sheet, e = endothelium, s = self-assembled stromal matrix, f = corneal fibroblasts. Scale bars, B) 50 μm, C-E) 20 μm.

Tissue-engineered posterior corneas were clear and could be manipulated using fine forceps (Fig 1). Histology cross-sections of these posterior corneal substitutes showed a monolayer of endothelial cells adhered to the two-layer self-assembled stromal matrix (Fig 2B). The SHG and THG signals were very strong across the entire thickness of the specimens and could both be acquired in epidetection. SHG acquisition delineated the two stromal sheets and provided sufficient spatial resolution to detect small regions of microscopic detachment between the two sheets (Fig 2C). THG signals imaged the endothelium as a uniform layer and revealed the stromal keratocytes between and under the two sheets (Fig 2D). The merged SHG/THG images showed an overall tissue structure very similar to that observed on the histology cross-sections (Fig 2E).

Thickness measurements made by SHG/THG microscopy and histology cross-sections differed significantly. SHG/THG measurements of the three fresh tissue-engineered posterior corneas yielded a mean (± SD) thickness value of 72 ± 38 μm, while the three paired posterior corneas measured on histology cross-sections (after fixation in formaldehyde and dehydration) yielded a mean thickness value of 37 ± 24 μm. The sample without endothelium was also measured with both techniques and yielded thickness values of 27 ± 4 μm and 23 ± 4 μm for histology and SHG/THG, respectively (Table 1).

**Table 1:** Thickness of the tissue-engineered posterior corneas, with and without endothelial cells, measured on histology cross-sections and with SHG-THG microscopy.

	Thickness measured on histology cross sections (μm)	Thickness measured with SHG/THG microscopy (μm)
**Engineered cornea 1 with endo**	38 ± 5	70 ± 5
**Engineered cornea 2 with endo**	61 ± 16	110 ± 10
**Engineered cornea 3 with endo**	14 ± 2	35 ± 9
**Engineered cornea without endo**	27 ± 4	23 ± 4

(Mean ± SD)

### En-face observations of the self-assembled stromal matrix

**Fig 3:**
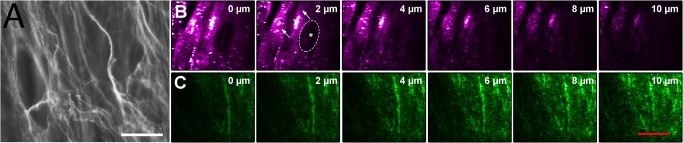
*En-face* observations of the self-assembled stromal matrix at different depths. A) Immunofluorescent staining of type I collagen. B) Sequence of six consecutive THG *en-face* images separated by 2 μm. Interpretation suggests the imaging of cells, with cytoplasm (maximal signal; see arrows) and putative silent nucleus (judging from shape and size) (dashed oval + asterisk). C) Corresponding SHG *en-face* images. Scale bars, 20 μm.

Traditionally, for *en-face* observation of type I collagen, an immunofluorescent staining is performed on fixed tissue. Fig 3A shows an immunofluorescent detection of type I collagen in the formaldehyde-fixed self-assembled stromal matrix. Collagen bundles are loosely packed and mostly aligned. A sequence of consecutive *en-face* images for SHG/THG microscopy in transmission is shown in Fig 3B and 3C. In these figures, SHG revealed a fibrillar network very suggestive of a stromal collagen network. Judging by shape, size, and distribution, THG images also suggested the presence of elongated stromal fibroblasts, with a strong THG signal captured at the level of the fibroblast’s cytoplasm [17, 25, 34]. Their putative nucleus, which measured about 10 μm [31], did not generate third harmonic signal, as expected for THG microscopy [35]. Fig 3B and 3C also illustrate the distribution of the cells within the collagen network.

**Fig 4:**
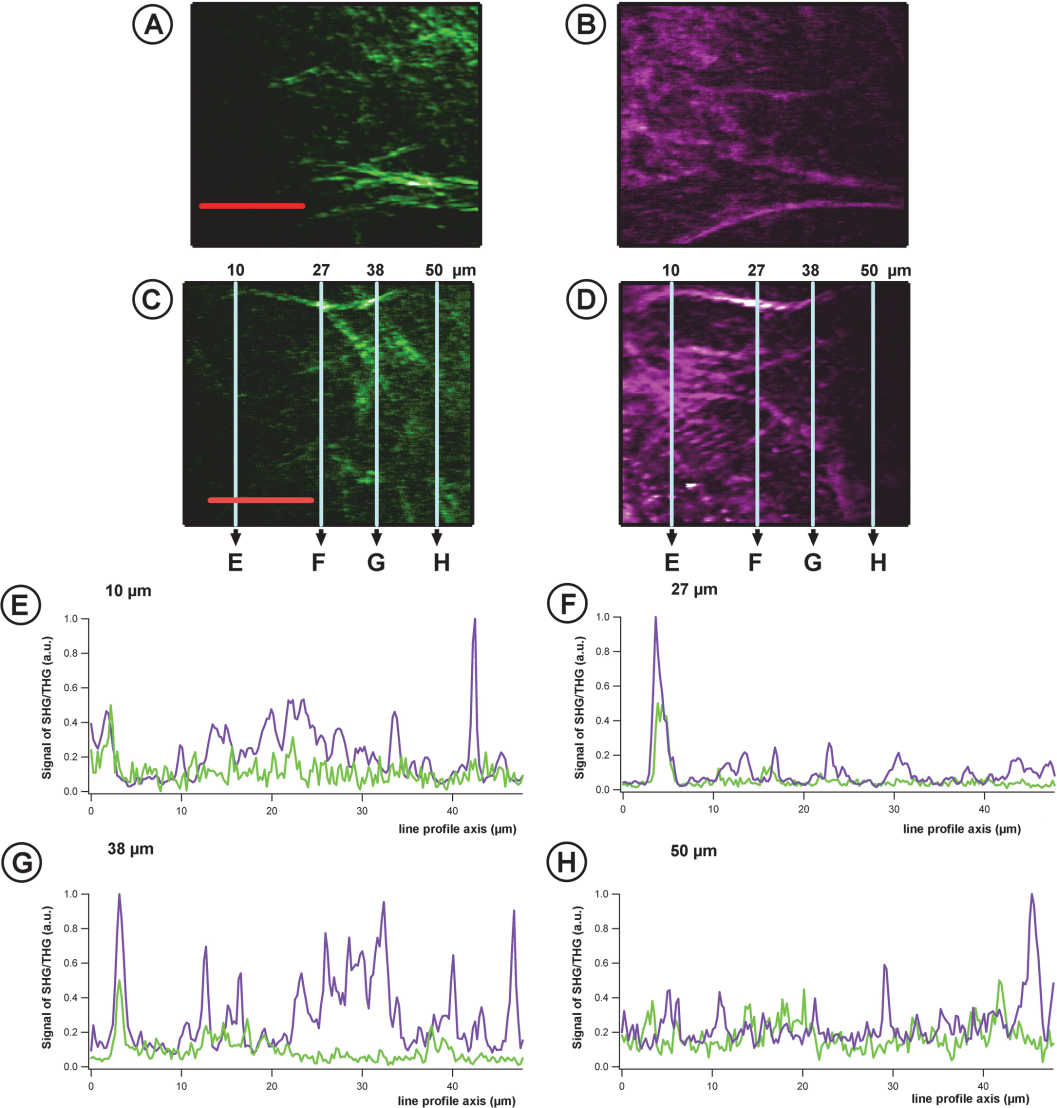
En-face observations of stromal collagen network of a posterior cornea with and without endothelium. A-B) “En face” SHG (A)/THG (B) images of a reconstructed posterior cornea with endothelial cells. C-D) “En face” SHG (C)/THG (D) images of reconstructed stroma without endothelial cells. E-H) Corresponding 1D graph of SHG (green line)/THG (purple line) intensity, extracted from line profiles at different locations in the images C and D (10, 27, 38 and 50 μm). Scale bars, 20 μm.

In order to evaluate if the presence of endothelial cells affected the structure of the engineered collagen stroma, acquisitions were also done in the stroma, for both engineered corneas (with and without endothelial cells) (Fig 4). Fig 4A and 4B were acquired in a posterior cornea with endothelial cells and Fig 4C to 4H in a posterior cornea without endothelial cells. A similar collagen architecture was observed in all corneas.

### En-face observation of the reconstructed corneal endothelium

**Fig 5:**
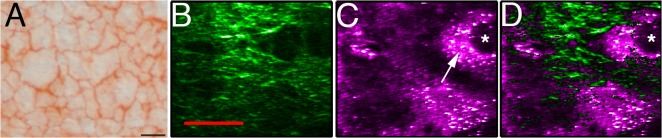
*En-face* observations of the endothelium. A) *En-face* observation of the endothelium stained with alizarin red; B) SHG imaging. C) THG imaging. D) Merge of SHG (green) and THG (purple). THG allowed to identify the cytoplasm (arrow) and the nucleus (asterisk) of the cells within the endothelial layer. The engineered tissue immersed in balanced salt solution is not perfectly flat, which explains why stroma and endothelial cells could be observed in the same focal plane.(D). Scale bars, A) 50 μm, B-D) 20 μm.

Alizarin red staining is commonly used to visualize the corneal endothelial surface. This stain accumulates at cell-cell junctions. Fig 5A shows an engineered endothelium stained with alizarin red. The endothelial cells cover the entire surface of the tissue-engineered stroma. Fig 5C and 5D show THG imaging of the endothelial cells, with a strong signal captured at the level of the cell cytoplasm and a very weak or absent signal at the level of the nucleus. The high signal-to-noise ratio of the SHG signal (Fig 5B and 5D), shows collagen in the same focal plane as that of the endothelial cells. This information is not provided by alizarin red stained *en-face* observations.

## Discussion

In this paper, we compared SHG/THG imaging with the histopathology methods traditionally used to characterize tissue-engineered corneas. The posterior cornea was engineered using primary human cells, which assembled their own extracellular matrix without the addition of exogenous biomaterials or scaffolds.

SHG/THG imaging offers major advantages over the traditional histopathology techniques. Essentially, it is a non-invasive technique that does not require tissue fixation and preserves the viability of the observed tissues. Indeed, the SHG/THG signals are not phototoxic in our set-up. At this excitation wavelength (1030 nm), there is no absorption (two-photon fluorescence) by endogenous chemical species [36] and by working at 50 mW with a numerical aperture of 0.9, the irradiance is completely compatible with in vivo studies [25]. Another advantage is that images are captured within a few minutes, eliminating the long, delicate and costly tissue processing steps. Furthermore, SHG/THG imaging allows the observation of whole tissues (contrary to histology which necessitates thin 5–10 μm-thick sections), along with keeping a high spatial resolution.

Another advantage of SHG/THG microscopy over traditional histology is the reliability of fresh tissue measurements, without the systematic under-estimation encountered with histology due to tissue shrinkage after fixation and dehydration [37]. In this study, the fresh tissue-engineered posterior corneas were on average 2.0-times thicker than those measured on the histology cross-sections. This difference in thickness was reproducible (1.8, 1.8 and 2.5-fold difference for samples 1, 2 and 3, respectively). Reliability of SHG/THG imaging to measure actual thickness of hydrated tissues is a major advantage over histology.

In this paper, the combined SHG/THG allowed global imaging of the tissue architecture (Fig 2), with clear visualization of the different layers, including the two stromal sheets and the overlying endothelium, as histology would do. THG microscopy images were suggestive of the typical morphology of the stromal and endothelial cells. The cell cytoplasm emitted a strong THG signal. The heterogeneity of the refractive index and of the third-order nonlinear susceptibility in the cytoplasm is believed to be attributed to the numerous mitochondria and other organelles found in the cell cytoplasm [17, 25, 34]. In contrast, the cell nucleus appeared as a dark region, which has also been reported by others [35]. SHG microscopy was able to image the stromal structure and showed a loose network of collagen bundles, relatively well aligned between and around the cells. Although similar SHG microscopy observations have been made by other groups in the native corneal stroma [21, 38, 39], this papers is the first to image the collagen architecture of this engineered corneal stromal substitute.

The structure of native human corneas was investigated using SHG/THG imaging in a recent paper by Aptel et al. [39]. Differences between their results and those obtained in the present study can be explained by differences in the microscope setup, as well as in the nature of the tissue observed. **(1)** We believe that the largest difference comes from the fact that in the experiments by Aptel et al., the corneal buttons were “maintained between two 150 μm-thick glass coverslips to flatten the corneal surface”. Flattening the surface reduces scattering of the laser beam, thus resulting in better spatial resolution, as well as higher throughput of the SHG/THG signal. The numerical aperture of our objective was lower (NA 0.9) than what was used in the Aptel paper (NA 1.2). These two combined differences may modify the imaging outcome. **(2)** The Aptel paper imaged a native human cornea, the structure of which is different from that of a tissue-engineered cornea, with a well-organized compact collagen structure and tightly packed collagen fibrils of regular diameter and spacing. A tissue-engineered corneal stroma immersed in balanced salt solution is typically edematous, with irregular collagen spacing and distribution, as shown in a previous paper [40].

On a very practical point of view, there is an urgent need in tissue engineering for a non-invasive imaging technique that would allow safe monitoring during tissue production and validation prior to batch release, and SHG/THG microscopy is a new imaging technique that seems to respond very well to this need.

Interestingly, SHG/THG signals in this study pointed out islands of stromal collagen network within the endothelial cell mosaic (Fig 5). Since the tissue was immerged in a balanced salt solution to keep the cells alive, the surface was not perfectly flat. Thus, stroma and endothelial cells can be observed in the same focal plane. Another explanation could be that collagen fibrils are present between cells. This explanation is unlikely, because endothelial cells form a monolayer of cells that are bound to each other using tight junctions. Fig 5A clearly shows that endothelial cells form a monolayer of tightly packed cells. However, alizarin red coloration is limited to macroscopic examination of the tissue. It is thus possible that SHG/THG is able to reveal small regions of collagen fibrils in areas where endothelial cells would, for some reason, not be correctly bound to each other.

The newly secreted endothelial basement membrane, i.e. Descemet membrane, was not sufficiently thick at this stage of culture to separate the stromal and endothelial structures as it is the case in the native cornea. Since THG imaging is particularly relevant to the observation of Descemet membrane, as demonstrated previously [27], SHG/THG microscopy is quite an interesting tool to follow the development of Descemet membrane and the partition of the posterior layers within the tissue-engineered posterior cornea. Alizarin red staining cannot provide this type of information.

Many successive steps are required for the production of a tissue and it may take months before the final product can be analyzed. Thanks to its optical properties, SHG/THG microscopy can rapidly and efficiently image fresh tissues without affecting their integrity, which constitutes a powerful advantage in tissue engineering. The same tissue could thus be repeatedly analyzed throughout the entire engineering process.

New tissue engineering concepts can also be evaluated. For instance, culturing corneal fibroblasts on microstructured substrates allows a better alignment of the cells and type I collagen, giving rise to stronger and more transparent corneal stromal substitutes [31]. Alignment was demonstrated using type I collagen immunofluorescent staining and transmission electron microscopy [31]. With SHG/THG microscopy, the structure of these aligned stromas would have been documented more thoroughly.

Another advantage of SHG/THG microscopy over traditional histology is the reliability of tissue measurements, without the shrinkage effect of fixation and dehydration. In this study, the fresh tissue-engineered posterior corneas were 2.0-times thicker than on histology cross-sections.

SHG/THG microscopy allows tissue visualization both in cross-section and *en-face*. It can provide three-dimensional imaging of collagen network and cells. Successive tissue reconstructions may also allow video monitoring of the evolving tissue structures (deposition of collagen, etc.). These tools are not available with traditional microscopy. In the present study, our optical set-up provided a precise high magnification observation of a few cells (keratocytes or endothelial cells). Other optics could also be implemented to obtain a larger field of view, which would allow endothelial cell counts and morphometric analyses.

At this stage, SHG/THG imaging cannot completely replace the traditional methods used for characterizing engineered corneas, as it cannot identify specific proteins important for the assessment of corneal endothelial functionality, such as the Na^+^/K^+^-APTAse pumps. Furthermore, in the present study, SHG/THG imaging could not discriminate between different fibrillar collagen types. Even if type I is the main collagen form, as immunostaining shows, types III and V are also present in a corneal stroma. Progress is being made for the refinement of fibrillar collagen detection by polarization-resolved SHG microscopy [41] on the basis of their tensor elements so that future developments may eventually permit characterization of the engineered tissue without the need for immunofluorescence.

## Conclusion

The combination of SHG and THG imaging proved to be an efficient tool for the noninvasive imaging of tissue-engineered posterior corneas. THG allowed visualization of the cells, endothelial cells in particular, with a higher level of details than histology, immunofluorescent labeling and alizarin red staining. Collagen extracellular matrix of the stroma can be revealed by SHG. SHG/THG imaging offers several significant advantages over these traditional techniques. It allows high spatial resolution full thickness and *en face* imaging of fresh tissues, without the need for tissue fixation, dehydration, staining, and cutting. SHG/THG microscopy offers the unique advantage of allowing safe and continuous monitoring of the development of the different tissue layers during production. It allows, for instance, the observation of Descemet membrane deposition over time, a corneal layer clearly identified by THG at the interface between the stroma and the endothelium.

## Supporting Information

S1 Fig Measurement of the reconstructed cornea by SHG/THG microscopy. 1D graph of SHG/THG intensity across the thickness of sample presented in Fig 3B and 3C, showing the measurement method from point “A” to point “B”.
(TIF)
