# Supplementary figures and images for: Characterization of Tissue-Engineered Posterior Corneas Using Second- and Third-Harmonic Generation Microscopy
Source: PLoS One. 2015 Apr 28;10(4):e0125564. doi: 10.1371/journal.pone.0125564 (PMC4412819; doi:10.1371/journal.pone.0125564)

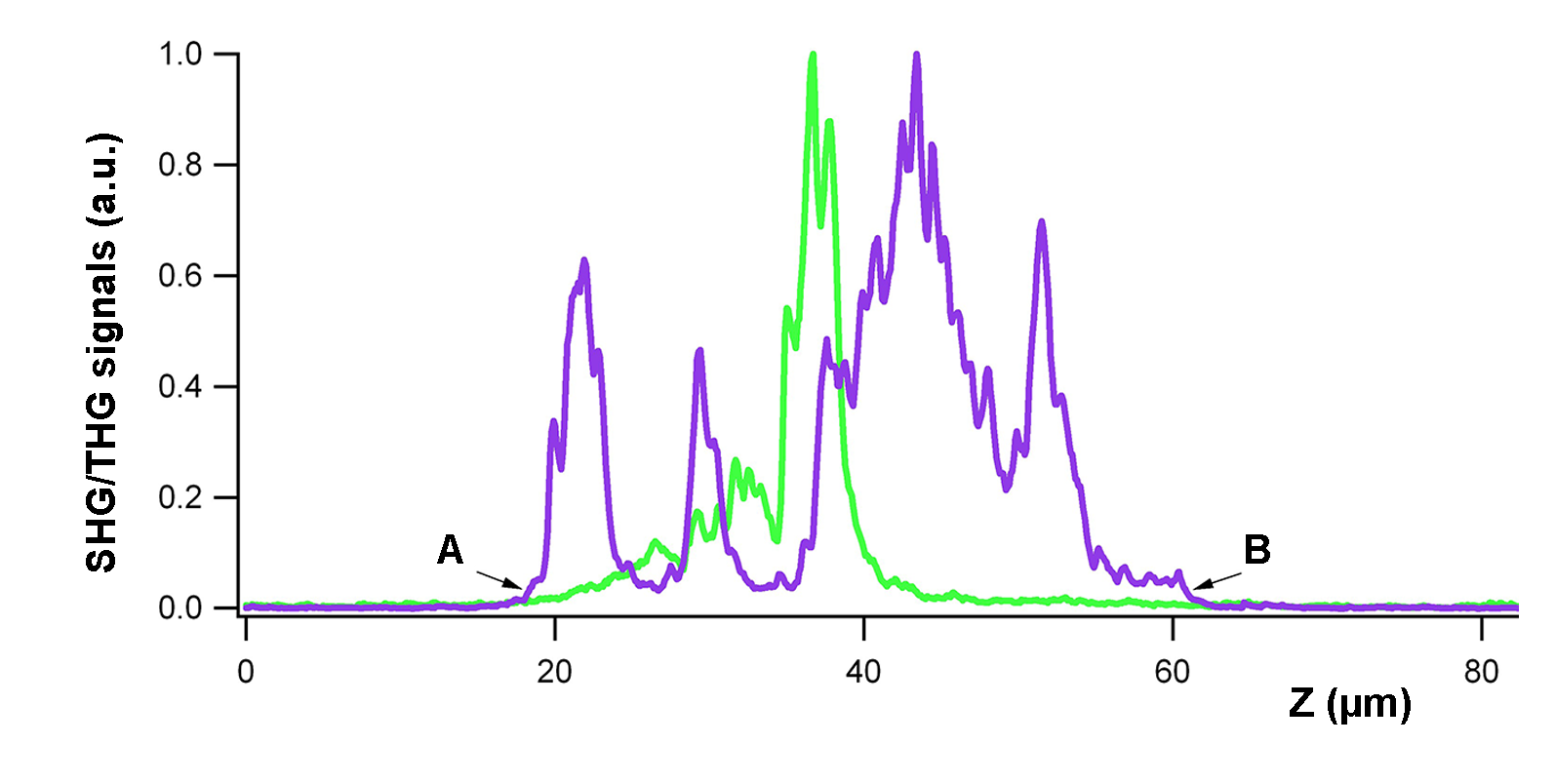

Supplement: S1 Fig — 1D graph of SHG/THG intensity across the thickness of sample presented in Fig 3B and 3C, showing the measurement method from point “A” to point “B”. (TIF) [file pone.0125564.s001.tif]
